# Synthesis, bioactivity, 3D-QSAR studies of novel dibenzofuran derivatives as PTP-MEG2 inhibitors

**DOI:** 10.18632/oncotarget.16595

**Published:** 2017-03-27

**Authors:** Ying Ma, Hui-Yu Wei, Yu-Ze Zhang, Wen-Yan Jin, Hong-Lian Li, Hui Zhou, Xian-Chao Cheng, Run-Ling Wang

**Affiliations:** ^1^ Tianjin Key Laboratory on Technologies Enabling Development of Clinical Therapeutics and Diagnostics (Theranostics), School of Pharmacy, Tianjin Medical University, Tianjin, China; ^2^ Eye Hospital, Tianjin Medical University, School of Optometry and Ophthalmology, Tianjin Medical University, Tianjin, China

**Keywords:** PTP-MEG2, dibenzofuran, synthesis, 3D-QSAR, docking

## Abstract

PTP-MEG2 plays a critical role in the diverse cell signalling processes, so targeting PTP-MEG2 is a promising strategy for various human diseases treatments. In this study, a series of novel dibenzofuran derivatives was synthesized and assayed for their PTP-MEG2 inhibitory activities. 10a with highest inhibitory activity (320 nM) exhibited significant selectivity for PTP-MEG2 over its close homolog SHP2, CDC25 (IC_50_ > 50 μM). By means of the powerful “HipHop” technique, a 3D-QSAR study was carried out to explore structure activity relationship of these molecules. The generated pharmacophore model revealed that the one RA, three Hyd, and two HBA features play an important role in binding to the active site of the target protein-PTP-MEG2. Docking simulation study indicated that 10a achieved its potency and specificity for PTP-MEG2 by targeting unique nearby peripheral binding pockets and the active site. The absorption, distribution, metabolism and excretion (ADME) predictions showed that the 11 compounds hold high potential to be novel lead compounds for targeting PTP-MEG2. Our findings here can provide a new strategy or useful insights for designing the effective PTP-MEG2 inhibitors.

## INTRODUCTION

Protein tyrosine phosphatases (PTPs) have been hot topics of research in biomedical science for the past two decades, and a number of PTPs have been involved in various human diseases, such as diabetes, autoimmune, cancer, and neurological disease [1–3]. Thus, the PTPs are now known as novel platforms for therapeutic intervention in human disease [4, 5].

Protein tyrosine phosphotase Meg2 (PTP-MEG2), an intracellular phosphatase belonging to the PTPs family, was originally cloned from human MEG-01 megakarocyte and umbilical vein endothelial cell cDNA libraries [6]. It is widely expressed in brain, leukocytes, endocrine, and exocrine cells and located on the cytoplasmic face of secretory vesicles [6, 7]. The enzyme is composed of two domains, namely catalytic domain and Sec14p homology domain. The catalytic domain located at the C-terminus has a sequence identity of about 30–40% in the catalytic domains with other known PTPs; while the other non-catalytic domain displays 24–29% sequence identity to cellular retinal dehyde-binding protein (CRALBP), α-tocopherol transfer protein, and yeast Sec14p [8, 9]. Molecular biology and genetic studies have shown that PTP-MEG2 plays a critical role in the diverse cell signalling processes [6, 10–14]. Owing to the highly homologous to Sec14p, which acts as a phosphatidylinositol transfer protein through the Golgi complex, PTP-MEG2 may also play a significant role in regulating the transfer of lipid molecules [15]. Moreover, the expression of PTP-MEG2 is elevated in polycythemia vera erythroid progenitor cells and is essential for growth and expansion of erythroid cells [16]. In addition, studies demonstrated that PTP-MEG2 inhibited insulin-induced phosphorylation of the insulin receptor, and depletion of PTP-MEG2 in the diabetic mice enhanced the insulin sensitivity, suggesting that it acts as a mediator of blood glucose homeostasis which in turn may be an effective drug target for treating type2 diabetes [17]. Furthermore, it promotes intracellular secretary homotypic vesicle fusion in hematopoietic cells, and dephosphorylation of epidermal growth factor receptor (EGFR) and ErbB2 resulted in the impaired activation of Signal transducer and activator of transcription 3 (STAT3) and Signal transducer and activator of transcription 5 (STAT5) in breast cancer cells [18, 19]. Taken together, these data suggest that targeting PTP-MEG2 is a promising strategy for various human diseases treatments.

Unfortunately, PTP-MEG2 presents several key challenges in drug development due to the highly conserved PTP active sites which makes it difficult to discover compounds that could selectively inhibit single PTP protein, and the positively charged PTP-MEG2 active site which makes it tough to discover drugs that could get through the cell [20]. Despite these challenges, selective PTP-MEG2 inhibitor drug discovery could serve not only as chemical probes to understand how the normal physiology and pathological conditions controlled by tyrosine phosphorylation, but also as novel drugs for human diseases.

Until recently, one PTP-MEG2 inhibitor-compound7 had been developed, which could augment insulin signaling and enhance the insulin sensitivity and glucose homeostasis in diet-induced obese mice [20]; Wang group reported that compounds 4a and 4b inhibited PTP-MEG2 activity with an IC_50_ of 3.2 μM and 4.3 μM, respectively, which showed modest selectivity against protein tyrosine phosphatase 1B (PTP1B) and T cell protein tyrosine phosphatase (TCPTP) [21]. Dibenzofurans and derivatives are mainly biosynthesized by lichens and ascomycetes [22]. To the best of our knowledge, many reports have been dedicated to study the biological activities of dibenzofurans on usnic acid and more specifically its cytotoxic and antibacterial activities [23]. However, owing to their low abundance in nature, other derivatives remained less studied. Few studies published on biological activities of dibenzofurans as PTP-MEG2 inhibitors. In this study, some dibenzofurans derivatives were synthesized and assayed for their PTP-MEG2 inhibitory activities, hoping to discover some potential PTP-MEG2 inhibitors. In the present work we reported the synthesis of dibenzofuran derivatives with 3D pharmacophore study. The technique of CDOCKER was utilized to analyze the binding interactions between the inhibitors and PTP-MEG2 and the technique of ADME was used to evaluate the drugability of hit compounds in hoping that the findings thus obtained may validate the observed pharmacological properties and provide useful insights for developing novel and powerful drugs against human diseases.

## RESULTS AND DISCUSSION

### Chemistry

**Scheme 1:**
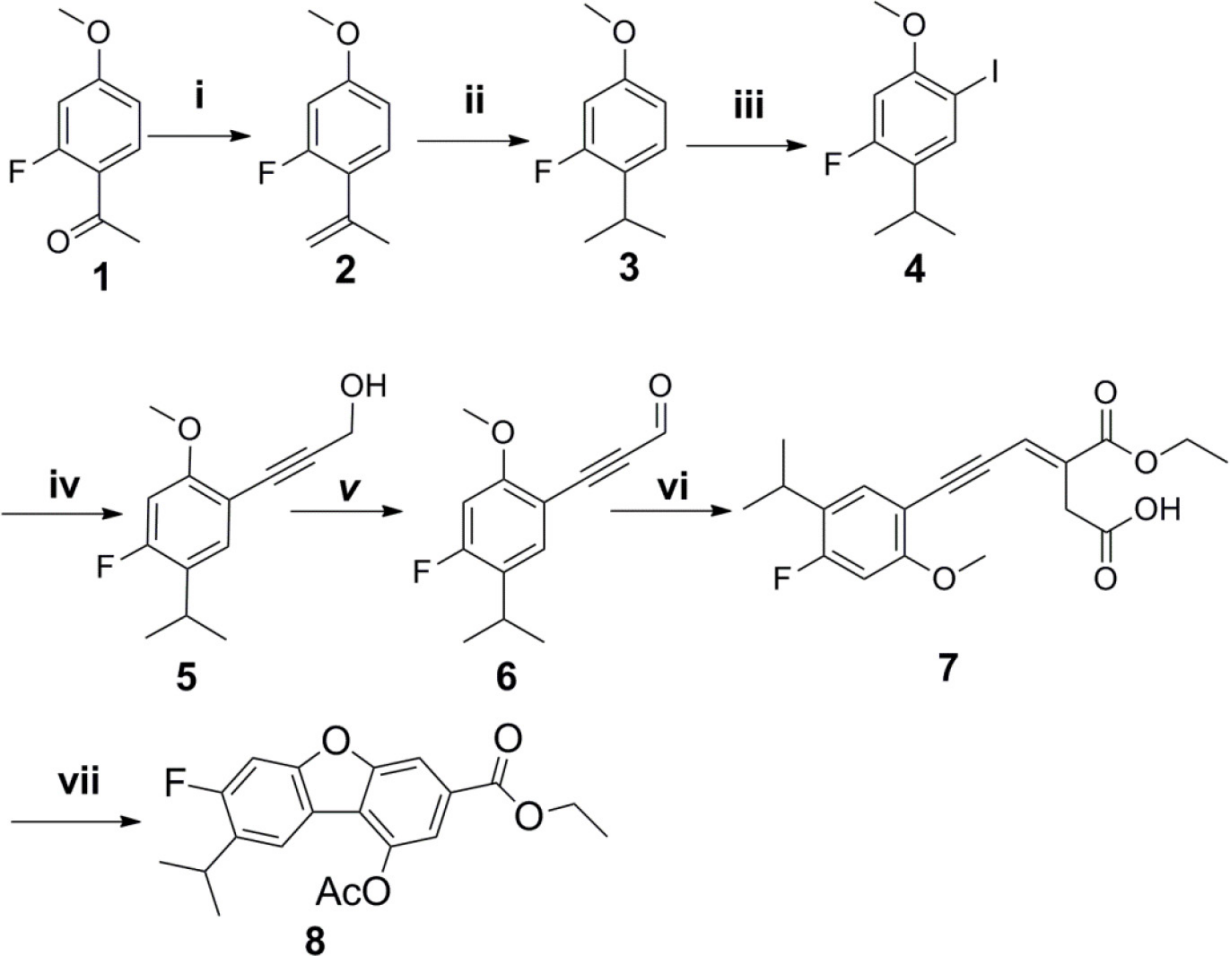
Reagents and conditions (**i**) n-BuLi, Ph3PCH3Br, THF, 96%; (**ii**) Pd/C, H2, 4 atm, MeOH, 91%; (**iii**) I2, Ag2SO4, MeOH, 90%; (**iv**) (Ph3P)2PdCl2, CuI, Et3N, THF, prop-2-yn-1-ol, 84%; (**v**) MnO2, DCM, 80%; (**vi**) Toluene, overnight, 67%; (**vii**) Ac2O,NaOAc, hydroquinone, reflux, 78%.

**Scheme 2:**
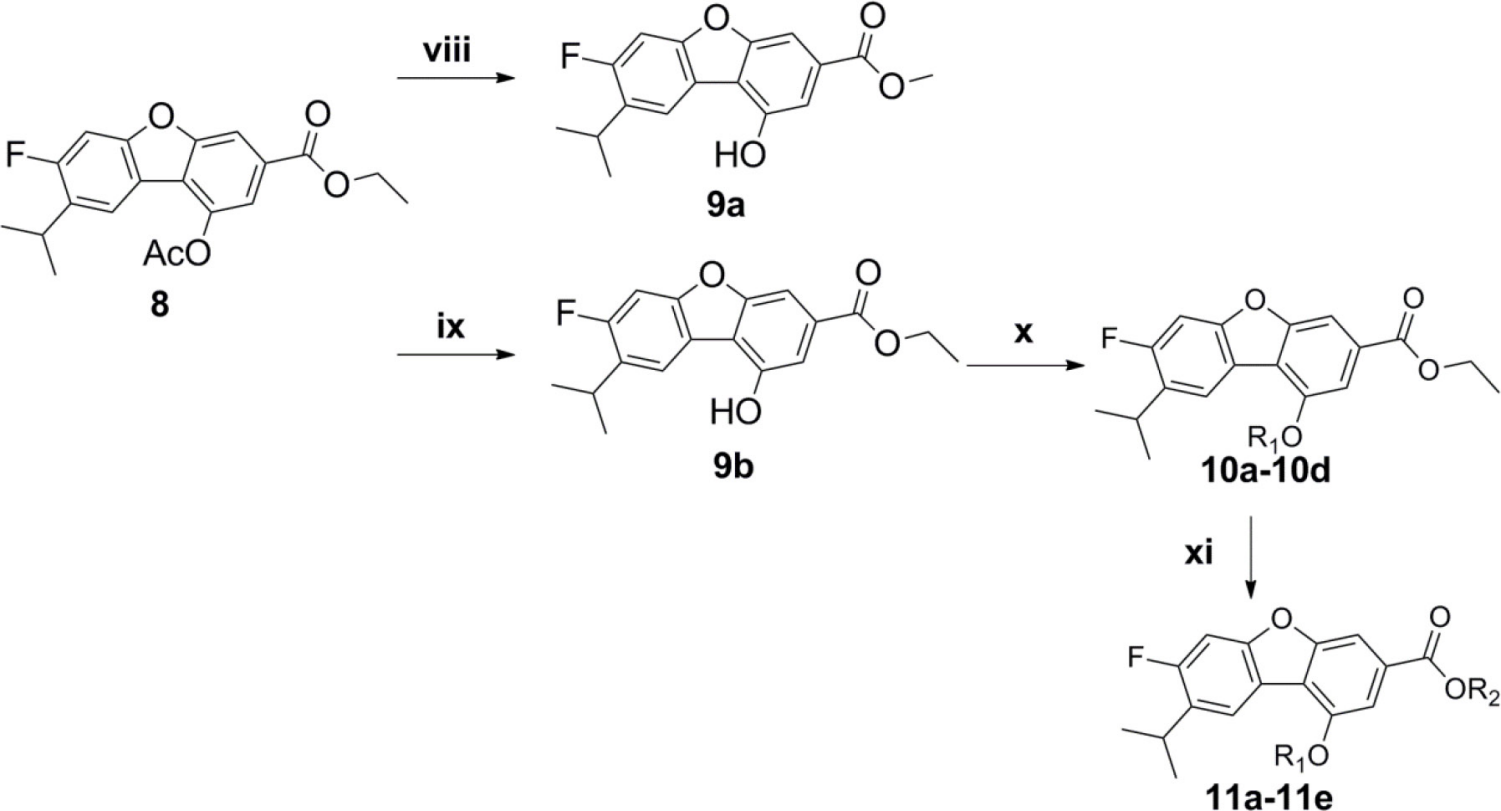
Reagents and conditions (**viii**) MeONa, MeOH, 62%; (**ix**) HNEt2, MeOH; (**x**) K2CO3, R1-Br, acetone; (**xi**) K2CO3, R2-Br, acetone. 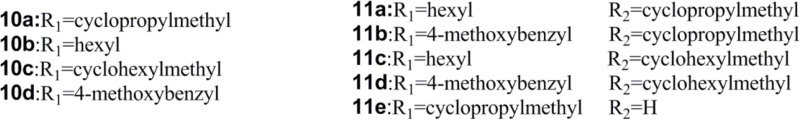

The synthetic strategy to prepare the target compounds is illustrated in Schemes 1–2. The carbon-carbon double bonds intermediate compound 2 was prepared from 1-(2-fluoro-4-methoxyphenyl)ethan-1-one and methyl triphenyl phosphonium bromide by Wittig reactions, followed by hydrogen reduction with Pd/C as catalyst under 4 atm of hydrogen to afford compound 3, which was iodinated with iodine catalyzed by silver sulfate to give compound 4. The key intermediate compound 5 was achieved through compound 4 and propargyl alcohol with Pd and Cu as catalyst by Sonogashira reaction [24]. Oxidization of compound 5 by manganese dioxide followed by Wittig reaction and cyclization gave compound 8 for two steps [25]. Subsequently, alcolization of compound 8 and amidation of compound 8 and then reaction of compound 9a and compound 9b with halohydrocarbon by Williamson reaction afforded analogues compound 10a-10d. Next hydrolyzation of compound 10a-10d with 2N NaOH aqueous solution followed by esters synthesis with halogenated hydrocarbon gave analogues compound 11a-11e.

The structures of all the newly synthesized compounds were characterized by ^1^H NMR, ^13^C NMR, ESI-MS.

### Biological evaluation

Table 1 listed the PTP-MEG2 inhibitory activities of the 11 dibenzofuran derivatives. It can be seen from Table 1 that most of these molecules exhibited mild inhibitory activities against human PTP-MEG2 with IC_50_ values at about 0.32–5.35 μM. 10a showed the most potent PTP-MEG inhibitory activity with the IC_50_ value at 0.32 μM.

**Table 1:** Structure and PTP-MEG2 inhibitory activity of dibenzofuran derivatives

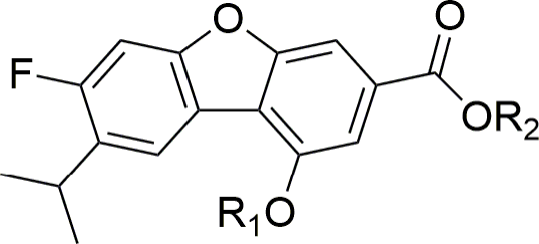							
Cpds	R_1_	R_2_	Fit values	Docking score	PTP-MEG2 IC_50_ (μM)	SHP-2 IC_50_ (μM)	CDC25 IC_50_ (μM)
8	CH_3_CO	ethyl	3.42	24.25	3.7 ± 0.22	> 50	> 50
9a	H	methyl	3.34	22.65	5.0 ± 0.40	> 50	> 50
10a	cyclopropylmethyl	ethyl	6.60	58.28	0.32 ± 0.02	> 50	> 50
10b	hexyl	ethyl	5.29	27.54	1.22 ± 0.11	> 50	> 50
10c	cyclohexylmethyl	ethyl	5.59	30.30	1.19 ± 0.08	> 50	> 50
10d	4-methoxybenzyl	ethyl	3.44	23.78	3.39 ± 0.34	> 50	> 50
11a	hexyl	cyclopropylmethyl	4.61	25.66	2.41 ± 0.17	> 50	> 50
11b	4-methoxybenzyl	cyclopropylmethyl	6.00	33.29	1.06 ± 0.10	> 50	> 50
11c	hexyl	cyclohexylmethyl	3.56	26.00	3.16 ± 0.32	> 50	> 50
11d	4-methoxybenzyl	cyclohexylmethyl	5.99	57.28	0.83 ± 0.04	> 50	> 50
11e	cyclopropylmethyl	H	3.01	17.82	5.35 ± 0.43	> 50	> 50

Each experiment was performed in triplicate. Data are presented as mean ± S.E.M.

All the molecules were substituted in part R_1_ and R_2_. The most active molecule 10a (R_1_ = cyclopropylmethyl, R_2_ = ethyl) and other molecules with suitable hydrophobic groups in these two positions (8, 10a, 10b, 10c, 10d, 11a, 11b, 11c, and 11d) were more active than the unsubstituted ones in either of the two places (9a and 11e). Besides, by comparison with the activities between compounds 10b-10d and 11a-11d, it was found that remaining bulky aromatic group (4-methoxybenzyl) at R_1_ and modifying at R_2_ revealed that increased steric bulk was preferred in the position R_2_ to improve the activity (the order of inhibition was 11d > 11b > 10d), whereas remaining hydrophobic group (hexyl) at R_1_ and modifying at R_2_indicated that increased steric bulk led to significantly decrease in the inhibitory activity (the order of inhibition was 11c < 11a < 10b). In addition, variation of the alkyl group at R_1_ and remaining small size alkyl group (ethyl) at R_2_ showed that decreased steric bulk at R_1_ would improve the PTP-MEG2 inhibitory activity. Consequently, the possible SARs of PTP-MEG2 inhibitors observed from the biological results is that compounds with appropriate hydrophobic and bulky substituents in parts R_1_ and R_2_ might acquire higher activities (10a, 10b, 10c, 11b, and 11d). The hypothesis will be tested in the following 3D-QSAR study.

### 3D pharmacophore studies

**Figure 1:**
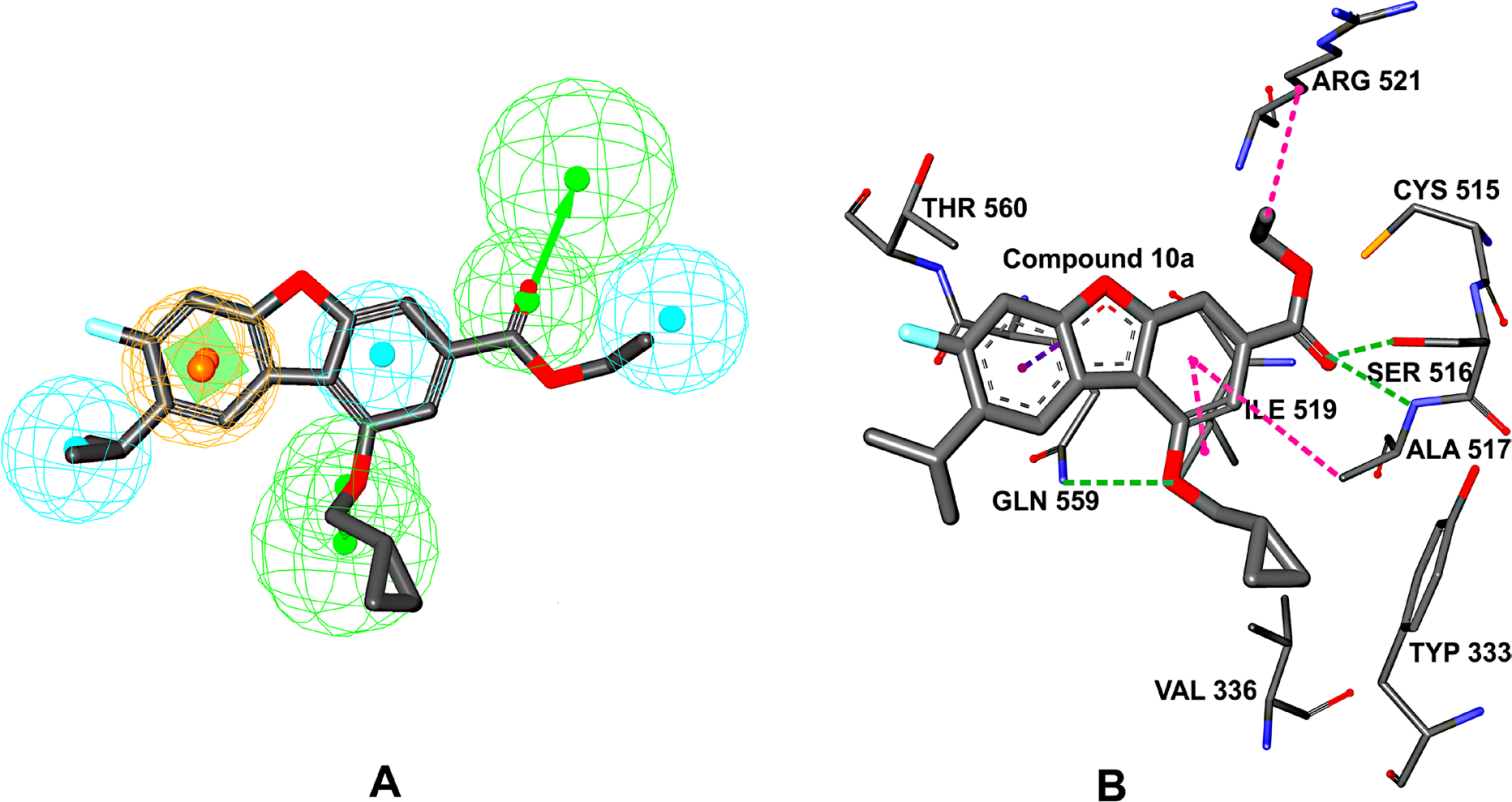
(**A**) Illustration to show the hypo3 generated by Hypogen. The best Hypogen model hypo-3-PTP-MEG2 mapped with Compound 10a. The features are colored coded with green, hydrogen-bond acceptor; cyan, hydrophobic; brown, ring aromatic. (**B**) Interaction of the receptor with the docked Compound 10a. The green dotted lines indicate the H-bond interactions of the receptor with Compound 10a. The purple dotted lines indicate the hydrophobic interactions of the receptor with Compound 10a.

We employed the HipHop module of Discovery studio v3.5 software to build reasonable 3D-common feature hypotheses. 10 optimal pharmacophoric hypotheses were created. As given in Table 2, the hypo1, hypo2, hypo5, hypo6, hypo9 and hypo10 have the same molecular features that contain two RA(ring aromatic), two Hyd (hydrophobic), and one HBA (hydrogen bond acceptor), while the hypo3, hypo4, hypo7 and hypo8 had the same molecular features that contained one RA, three Hyd, and two HBA with different 3D spatial arrangements. To validate the resulting models, we subjected our pharmacophores to ROC (receiver operating characteristic) analysis to assess their abilities to selectively capture diverse PTP-MEG2 inhibitors from a large list of decoys. The testing set included 3 active compounds and 90 decoys searched from zinc database [26]. The ROC testing set (93 compounds) was screened by each pharmacophore for ROC analysis. In ROC analysis, the ability of a particular pharmacophore model to distinguish a list of compounds as actives or inactives was indicated by the area under the curve (AUC) of the resulting ROC as well as other two parameters: sensitivity and specificity [27, 28]. Table 2 showed the ROC performances of our 10 optimal pharmacophores. As shown in Table 2, it can be concluded that hypo3 performed better than the other 9 pharmacophores based on ROC-AUC, sensitivity and specificity. The 3D-common feature pharmacophore-hypo3 (Figure 1) has been developed to derive the structure-activity relationships of PTP-MEG2 inhibitors. The generated 3D-common feature pharmacophore hypothesis containing one RA, three Hyd, and two HBA was applied to explain the pharmacophoric site specifications of the PTP-MEG2 inhibitory activities of dibenzofuran derivatives. The generated pharmacophore model revealed that the one RA, three Hyd, and two HBA features played an important role in binding to the active site of the target protein-PTP-MEG2. One RA and three Hyd features demonstrated the appropriate active shape of the molecule, displaying the required placement of aromatic moiety and hydrophobic group. Two HBA features at the given positions were vital in the molecule to bind to the target protein. As we can see from the pharmacophore, the essentials for the specification of PTP-MEG2 inhibitory activity of dibenzofuran derivatives are listed as follows: 1) the ring aromatic property of the fluoro-phenyl group in the fused ring system; 2) the hydrophobic property of the isopropyl group and the phenyl group in the fused ring system, and the ethyl moiety at R_2_; 3) the hydrogen bond acceptor property of carbonyl oxygen and oxygen atom in the alkyloxy group in the fused ring system. The mapping of 10a, as a representative, in hypo-3 was shown in Figure 1. As shown in Supplementary Figure 1, 10a and 11d mapped all the features in hypo-3, which might explain why 10a and 11d possessed higher potent activities than the other molecules. Interestingly, 10a had small size alkyl group at R_1_ and R_2_, while 11d possessed large steric bulk at R_1_ and R_2_. Although11d, 11b and 10d were substituted by same bulky aromatic group (4-CH_3_OOCphCH_2_) at R_1_, and 10b, 11a and 11c were substituted by same steric bulk group (hexyl) at R_1_, all of them mapped the features in hypo-3 in the same way. 11d hold a bulky aromatic group at R_2_, rather than having small size group at R_2_, such as 11b and 10d, possessed a better match with all the features in the model. However, 10b had small size alkyl group at R_2_, rather than having large size group at R_2_, such as 11a and 11c, possessed a better match with all the features in the model. 9a and 11e with the unsubstituted ones in either of R_1_ and R_2_ missed the Hyd feature. From the above, compounds with appropriate hydrophobic and bulky substituents in parts R_1_ and R_2_ would match with all the mapped common features in the anticipated model, which was consistent with the experimental data.

**Table 2:** HipHop-generated hypotheses and validation with known actives/inactives

Hypotheses	features	Rank	Total actives	Total inactives	True positives	True negatives	False positives	False negatives	Sensitivity	specificity	ROC
hypo1	RRHHA	102.706	3	90	2	73	17	1	0.6667	0.81111	0.706
hypo2	RRHHA	102.706	3	90	2	75	15	1	0.6667	0.83333	0.806
hypo3	RHHHAA	102.151	3	90	2	79	11	1	0.6667	0.87778	0.994
hypo4	RHHHAA	102.151	3	90	2	72	18	1	0.6667	0.80000	0.694
hypo5	RRHHA	101.727	3	90	2	76	14	1	0.6667	0.84444	0.883
hypo6	RRHHA	101.707	3	90	2	76	14	1	0.6667	0.84444	0.883
hypo7	RHHHAA	100.889	3	90	2	71	19	1	0.6667	0.78889	0.787
hypo8	RHHHAA	100.889	3	90	2	73	17	1	0.6667	0.81111	0.804
hypo9	RRHHA	99.090	3	90	2	76	14	1	0.6667	0.84444	0.804
hypo10	RRHHA	99.090	3	90	2	73	17	1	0.6667	0.81111	0.726

### Molecular docking

The model was obtained by docking ligand to the PTP-MEG2 domain (from PDB 4GE6) using the methods we have described in materials and methods section. The 10a, ranked the first in the fit value and in the PTP activity assay, inhibited the activity of PTP-MEG2 with an IC_50_ of 320 nM. 10a exhibited significant selectivity for PTP-MEG2 over its close homolog SHP2, CDC25 (IC_50_ > 50 μM). The preferred co-ordination mode of 10a is described in Figure 1. To assess the hypo3-PTP-MEG2, we compared the pharmcophore model with the active site of PTP-MEG2. The hypo3-PTP-MEG2 model consists of one RA, three Hyd, and two HBA (Figure 1A). The HBAs are oriented to interact with the nucleophilic catalytic residues: Ser 516, Ala 517, and Gln 559. The Hyd is pointed towards Ala517, Ile 519 and Arg521 and the RA is oriented to interact with Gln 559. A close-up view for the protein-ligand interactions at the binding pocket thus defined is shown in Figure 1B. The results of receptor–ligand interactions obtained from the docking simulation had proved that the key residues for the binding interactions between 10a and the receptor were fully consistent with the previous reports [20]. 10a is found in the PTP-MEG2 active-site pocket and forms extensive interactions with residues in the P-loop (residues 514−521), the pTyr recognition loop (residues 331−338), and the Q-loop (residues 558−564). The O19 atom of 10a makes two hydrogen bonds [29] with the main chain amides Ser516 and Ala 517of the P-loop; The O21 atom of 10a also forms one hydrogen bond with Gln559 of the Q-loop. In addition to the polar interactions, the dibenzofuran group participates in hydrophobic interactions with Ile519, Ala517 in the P-loop and Gln559 in the Q-loop. The dibenzofuran group is involved in pi-sigma hydrophobic interaction [30] with Gln559 and two pi-alkyl hydrophobic interactions with Ala517 and Ile 519. The C25 atom of 10a is engaged in alkyl hydrophobic interaction [31] with Arg521 in Q-loop. The 2D diagram of PTP-MEG2-10a, interactions were shown in Supplementary Figure 2, pink plates such as Cys515, Ser516, Ala517, Gly518, Ile519,Gly520, Arg521, Gln559, Gln563 were involved in hydrogen bonding, charge or polar interactions, while green plates like Tyr307, Arg311, Tyr333, Asp335, Val336,Lys411, Thr522, Thr560, Pro561 represented van der waals interactions. Interestingly, Pro561 is unique to PTP-MEG2, which means no other PTPs have the same amino acids at the corresponding positions. It is likely that the van der waals interactions between dibenzofuran group were responsible for the potency and selectivity of 10a. Collectively, the structural observations offered direct evidence that 10a achieved its potency and specificity for PTP-MEG2 by targeting unique nearby peripheral binding pockets as well as the active site.

### ADME

Some molecular properties of the dibenzofuran derivatives such as the AlogP, molecular weight, number of aromatic ring, number of H-acceptors, number of H-donors, number of rings, number of aromatic rings, number of rotatable bonds, molecular fraction polar surface area were calculated by “Calculate Molecular Properties” module of the Discovery Studio v3.5. Some pharmacokinetic properties of these derivatives such as PSA, Solubility, human intestinal absorption, blood brain barrier, cytochrome p450 2D6, protein binding, and hepatotoxicity plasma were also predicted by Discovery Studio v3.5. The results thus obtained are listed in the Tables 3 and 4, respectively. Results of pharmacokinetic screening indicated that 8, 9a, 10a, 10c, 11b, 11c, 11d, 11e followed the Lipinski's rule of five for oral bioavailability. Human Intestinal Absorption (HIA) and solubility are two key factors that affect oral bioavailability. Without moderate to high intestinal absorption, the therapeutic effect of drugs can appreciably diminish. Solubility has a pronounced effect on the pharmacological activity of a compound in terms of its uptake, distribution, and ultimately bioavailability. The compound 10b, 10c, 11a and 11c showed lipophilic nature due to high Log*P* value, while compound 11d showed both high lipophilicity and low human intestinal absorption due to high LogP and molecular weight. CYP2D6 is responsible for the metabolism and elimination of approximately 25% of clinically used drugs. The inhibition of CYP2D6 by a drug constitutes the majority cases of drug-drug interaction. Ten compounds were predicted to be non-inhibitors of cytochrome P450 2D6 (CYP2D6), which is one of the important enzymes involved in drug metabolism. The predicted plasma protein binding parameter is an important parameter for drug distribution. All compounds were found to be highly bound with plasma protein. For hepatotoxicity, nine compounds were predicted non-toxic. For brain/blood barrier, compound 10a had a good penetrant level, and three compounds had a moderate penetrant level. Therefore, as mentioned above, the values for the ADME properties of compound 10a, 10c, 11b, 11c, and 11d listed in Table 4 are within the acceptable range for human beings, indicating these compounds found in this study can be utilized as candidates for the purpose of developing new drugs.

**Table 3:** Molecular properties for the dibenzofuran derivatives

	TPSA	Num H_Donors	Num H_Acceptors	Num RotatableBonds	Num Rings	Num AromaticRings	Lipinski rules	Molecular_weight
8	65.74	0	4	6	3	3	pass	358.36
9a	59.67	1	3	3	3	3	pass	302.297
10a	48.67	0	3	7	4	3	pass	370.14
10b	48.67	0	3	10	3	3	failed	400.483
10c	48.67	0	3	7	4	3	pass	412.494
10d	57.9	0	4	8	4	4	failed	436.472
11a	48.67	0	3	11	4	3	failed	426.52
11b	57.9	0	4	9	5	4	pass	462.509
11c	48.67	0	3	11	4	3	pass	468.6
11d	57.9	0	4	9	5	4	pass	504.589
11e	62.5	0	3	5	4	3	pass	342.361

**Table 4:** The ADME prediction for the dibenzofuran derivatives

	ALogP^a^	Solubility-level^b^	BBB-Level^c^	CYP2D6 Prediction	Hepatotoxic# Prediction	Absorption-level^d^	PPB# Prediction	PSA_2D
8	4.705	2	1	False	True	0	True	65.016
9a	4.346	2	1	False	True	0	True	59.6
10a	5.741	1	0	False	False	0	True	47.715
10b	7.161	0	4	True	False	3	True	47.715
10c	7.109	0	4	False	False	3	True	47.715
10d	6.488	1	4	True	False	1	True	56.645
11a	7.633	0	4	True	False	3	True	47.715
11b	6.959	1	4	False	False	2	True	56.645
11c	9.001	0	4	False	False	3	True	47.715
11d	8.328	0	4	False	False	3	True	56.646
11e	5.166	2	1	False	True	0	True	56.085

a:AlogP, the logarithm of the partition coefficient between n-octanol and water; b:Aqueous solubility level: 0 (extremely low); 1 (very low, but possible); 2 (low); 3 (good);c: BBB level: 0 (very good); 1 (good); 2 (moderate); 3 (poor);4(undefined) ; d: Human intestinal absorption level: 0 (good); 1 (moderate); 2 (poor); 3 (very poor).

## CONCLUSIONS

The goal of this study was to synthesize a series of dibenzofuran derivatives and evaluate the PTP-MEG2 inhibitory activities of these compounds. 3D-QSAR study using HipHop methods was applied to study the structure-activity relationship. The best hypothesis contains one RA, three Hyd, and two HBA. The compounds with appropriate hydrophobic and bulky substituents in parts R_1_ and R_2_ would match with all the mapped common features in the anticipated model. It is interesting to discover that 10a exhibited significant selectivity for PTP-MEG2 (320 nM) over its close homolog SHP2, CDC25 (IC_50_ > 50 μM). Through molecular docking, a most likely binding mode was proposed, suggesting that the potency and selectivity of the PTP-MEG2 inhibitors could be achieved by targeting peripheral pockets and the active site. It was further validated by the outcomes of their ADME predictions that the new inhibitors hold high potential to become drug candidates. Or at the very least, our 3D QSAR model can be useful and predictive tool to develop novel PTP-MEG2 inhibitors.

## MATERIALS AND METHODS

### Chemistry

#### General

All the reagents were purchased from commercial suppliers and were used without further purification unless otherwise indicated. All the reactions were monitored by thin-layer chromatography (TLC) on silica gel precoated F254 Merck plates, and spots were examined under UV light (254 nm). All column chromatography was performed using 200-300 mesh silica gel. ^1^H NMR and^13^C NMR spectra were taken on a Bruker Avance 300-MHz NMR Spectrometer at 300 K with TMS as the internal standard, and CDCl_3_ and DMSO-d_6_ were used as solvent, the values of the chemical shifts (δ) are expressed in parts per million (ppm), and coupling constants (J) are expressed in hertz (Hz). MS spectra were recorded on an Agilent 1100 LC/MSD (ESI) Mass Spectrum.

### *General method I*: Williamson ether synthesis reaction

To a well stirred solution of compound 9b (0.1 g, 1 mmol) in anhydrous acetone, was added Cesium Carbonate (Cs_2_CO_3_) (0.63 g, 2 mmol) and (bromomethyl)cyclopropane (0.1 g, 2 mmol), the mixture was heated at reflux overnight under N_2_ atmosphere anhydrous when most of the starting materials were converted into the target compound. The mixture was filtrated over a pad of celite and washed with chloroform. The precipitated product was collected by filtration, and further purified by silica gel column chromatography with 10%~12% ethyl acetate in petroleum ether as elute to afford the final product.

### *General method II*: Carboxylic acid ester hydrolysis reaction

A mixture of carboxylic acid ester derivatives (0.15 mmol) and 2N NaOH aqueous solution (10 mL) in MeOH (10 mL) was stirred at ambient temperature overnight. TLC and LC-MS examination showed that most of the starting materials were converted into the target compound. After the reaction, the mixture was acidified to pH 2 with 1N HCl aqueous solution. Subsequently, the crude product was washed with water (2 × 10 mL), and was air-dried to give crude product.

### *General method III*: Esters synthesized reaction

To a well stirred solution of carboxylic acid ester derivatives (0.15 mmol) in acetone (20 mL) was added halogenated hydrocarbon (0.15 mmol) and Cs_2_CO_3_ (0.30 mmol). The result mixture was heated at reflux until most of the carboxylic acid ester derivative was converted into the target compound. Then, the mixture was separated with a funnel and aqueous phase was extracted with ethyl acetate. The combined organic phases were washed with brine and dried over anhydrous Na_2_SO_4_. After filtration and concentration, the residual was purified by column chromatography (200–300 mesh silica gel, 10~12% ethyl acetate in PE).

### 2-fluoro-4-methoxy-1-(prop-1-en-2-yl) benzene (2)

Under N_2_ atmosphere, to a solution of methyl triphenyl phosphonium bromide (110 g, 310 mmol) in dry tetrahydrofuran (THF) (550 mL) was added BuLi (0.25 mL, 2.5 M solution in THF) dropwise at −65°C and the reaction stirred for 30 min. Then 1-(2-fluoro-4-methoxyphenyl)ethan-1- one (40 g, 238 mmol) in dry THF (100 mL) was added dropwise and the reaction was stirred at −65°C for 1.5 h. Then, the reaction mixture was naturally heated to room temperature and stirred at room temperature overnight. TLC and LC-MS examination showed that most of the starting material was converted into the target compound. Acetic acid was introduced into the system with stirring to quench the reaction, which was extracted with ethyl acetate (150 mL) (× 3), and washed with water (× 2) and saturated brines (× 2), dried over anhydrous MgSO_4_ and filtered and concentrated *in vacuo* to give the crude product. Purification by column chromn chromatography (200–300 mesh silica gel, 8%~20% ethyl acetate in PE) gave final product compound 2 (38 g, yield 96%).^1^H NMR(300 MHz, *d*_6_-DMSO) *δ*:7.26 (t, 1H), 6.81 (dd, *J* = 8.0, 2.0, 1H), 6.73 (dd, *J* = 8.0, 2.0, 1H), 5.17 (s, 2H), 3.75 (s, 3H), 2.05 (s, 3H).

### 2-fluoro-1-isopropyl-4-methoxybenzene (3)

After two vacuum/H_2_ cycles to replace air inside the reaction tube with hydrogen, the mixture of the compound 2 (38 g, 229 mmol) and10% Pd/C (2 g) in MeOH (250 mL) was vigorously stirred at room temperature under 4 atm of hydrogen for 6 h. The reaction mixture was filtered using a membrane filter (Millipore, MillexLH, 0.45 μm), and the filtrate was concentrated to provide the compound 3 as light yellow oil(35 g, yield 91%). The crude compound 3 was used without further purification. ^1^H NMR(300 MHz, CDCl_3_) *δ*:7.1 (t, 1H), 6.63 (dd, *J* = 8.0, 1.5, 1H), 6.56 (dd, *J* = 7.5, 1.5, 1H), 3.78 (s, 3H), 3.12 (m, 1H), 1.15 (m, 6H).

### 1-fluoro-4-iodo-2-isopropyl-5-methoxybenzene (4)

To a well stirred solution of the compound 3 (35 g, 208 mmol) in MeOH (200 mL) was added silver sulfate (65 g, 208 mmol), iodine (52 g, 208 mmol) and the reaction was stirred at room temperature for 6 h. TLC and LC-MS examination showed that most of the starting material was converted into the target compound. The solvent was removed by rotary evaporation and the solid was filtered through Büchner funnel and the filtrate was washed with MeOH (× 2). Purification by column chromn chromatography (200–300 mesh silica gel, 5%~10% ethyl acetate in PE) gave final product compound 4 (55 g, yield 90%). ^1^H NMR(300 MHz, CDCl_3_) δ: 7.57 (d, *J* = 9.6, 1H), 6.52 (d, *J* = 12.0, 1H), 3.88 (s, 3H), 3.12 (m, 1H), 1.21 (m, 6H).

### 3-(4-fluoro-5-isopropyl-2-methoxyphenyl)prop-2-yn-1-ol (5)

Under N_2_ atmosphere, to a solution of the compound 4 (35 g, 120 mmol) and propargyl alcohol(20 g, 360 mmol, 3 eq) in dry THF (1000 mL), and the mixture was cooled to 0°C with an ice-bath, was added copper(I) iodide (22.68 g,120 mmol, 1 eq) and dichlorobispalladium (70 mg, 0.1 mmol) stirred for 10 min. Then triethylamine (100 ml) was added dropwise and the reaction was stirred at room temperature for overnight. TLC and LC-MS examination showed that most of the starting material was converted into the target compound. Water was introduced to the system to quench the reaction, and the mixture was concentrated to remove most of the THF. The residual was extracted with ethyl acetate (2 × 50 mL) (× 2). The combine organic solution was washed with brine and dried over anhydrous MgSO_4_. Purification by column chromn chromatography (200–300 mesh silica gel, 10%~50% ethyl acetate in PE) gave final product compound 5 (22 g, yield 84%). ESI-MS: [M + NH_4_]^+^ = 240, ^1^H NMR(300 MHz, CDCl_3_) δ:7.28 (d, *J* = 8.4, 1H), 6.54 (d, *J* = 12.0, 1H), 4.54 (s, 2H), 3.85 (s, 3H), 3.08 (m, 1H), 1.21(m, 6H).

### 3-(4-fluoro-5-isopropyl-2-methoxyphenyl)propiolaldehyde (6)

A mixture of compound 5 (4.44 g, 20 mmol) and manganese dioxide (40 g) in dichloromethane (DCM) (100 mL) was stirred at ambient temperature for three days. TLC and LC-MS examination showed that most of the starting material was converted into the target compound. The reaction mixture was filtered using a membrane filter (Millipore, MillexLH, 0.45 μm), and the filtrate was concentrated to provide the compound 6 as colorless oil (0.65 g, yield 94%). The crude compound 6 was used without further purification. ESI-MS: [M + NH_4_]^+^ = 238,^1^H NMR(300 MHz, CDCl_3_) δ: 9.43 (s, 1H), 7.42 (d, *J* = 8.4, 1H), 6.59 (d, *J* = 12.0, 1H), 3.89 (s, 3H), 3.08 (m, 1H), 1.21 (m, 6H).

### (E)-3-(ethoxycarbonyl)-6-(4-fluoro-5-isopropyl-2-methoxyphenyl)hex-3-en-5-ynoic acid (7)

A mixture of compound 6 (3 g, 13.6 mmol) and 4-ethoxy-4-oxo-3-(triphenyl-l5-phosphanylidene) butanoic acid (5.5 g, 13.6 mmol)) in toluene (100 mL) was stirred at ambient temperature overnight. TLC and LC-MS examination showed that most of the starting material was converted into the target compound. The solvent was removed by rotary evaporation. Water and ethyl acetate were added into the reaction mixture and the organic layer was washed with brine and dried over anhydrous MgSO_4_. After filtration and concentration, the residual was purified by column chromatography (200–300 mesh silica gel, (ethyl acetate/PE/acetic acid, 10:100:1~30:100:1v/v/v). 3.20 g (yield 67%) of compound 7 was obtained as brown powder. ESI-MS: [M + H]^+^ = 349,^1^H NMR(300 MHz, CDCl_3_) δ: 7.29 (s, 1H), 7.06 (s, 1H), 6.52 (d, *J* = 12.0, 1H),4.24 (q, 2H), 3.86 (s, 3H), 3.77 (s, 2H), 3.08 (m, 1H), 1.31 (t, 3H), 1.21 (m, 6H).

### ethyl 1-acetoxy-7-fluoro-8-isopropyldibenzo[b, d]furan-3-carboxylate (8)

Under N_2_ atmosphere, to a well stirred solution of compound 7 (3 g, 8.6 mmol) in dry acetic anhydride (250 mL) was added sodium acetate (3 g, 8.6 mmol), hydroquinone (11 mg, 0.1 mmol), and the reaction was heated at reflux for 6 hours. TLC and LC-MS examination showed that most of the starting material was converted into the target compound. The solvent was removed by rotary evaporation. The residue was diluted with 50 mL of ethyl acetate. The mixture was washed with water and brine and dried over anhydrous Na_2_SO_4_. After filtration and concentration, the residual was purified by column chromatography (200–300 mesh silica gel, 10%~50% ethyl acetate in PE). Totally, 2.35 g (yield 78%) of target compound 8 was obtained.

ESI-MS: [M + NH_4_]^+^ = 376, ^1^H NMR(300 MHz, CDCl_3_) δ: 8.13 (s, 1H), 7.85 (s, 1H), 7.67 (d, *J* = 7.2, 1H), 7.27 (d, *J* = 7.2, 1H), 4.40 (m, 2H), 3.30 (m, 1H), 2.54 (s, 3H), 1.43 (t, 3H), 1.33(m, 6H),^13^C NMR(75 MHz, CDCl_3_) δ:168.44, 165.62, 162.71, 159.42, 157.18, 155.67, 144.30, 131.72, 129.20, 121.07, 120.05, 119.95, 117.60, 110.78, 99.48, 61.50, 27.45, 22.80, 21.01, 14.34.

### methyl 7-fluoro-1-hydroxy-8-isopropyldibenzo[b, d]furan-3-carboxylate (9a)

To a well stirred solution of compound 8 (1 g, 2.8 mmol) in MeOH (100 mL), was added sodium methoxide (3 g, 56 mmol) in MeOH(20 mL) in dropwise at 0°C with an ice-bath. The result mixture warmed to room temperature slowly and stirred until most of the compound 8 converted into the target compound 9a. After the reaction, the mixture was acidified to pH 1-2 with 5 mL acetic acid. The solvent was removed by rotary evaporation. The residue was diluted with 50 mL of ethyl acetate. The mixture was washed with water and brine and dried over anhydrous Na_2_SO_4_. The precipitated product was filtered, and purified by recrystallization from a mixed MeOH/H_2_O solution (MeOH:H_2_O; 3:1) to yield compound 9a (0.50g, yeild 59%).

ESI-MS: [M+NH_4_]^+^= 320, ^1^H NMR(300 MHz, CDCl_3_) *δ*: 8.01 (d, *J* = 7.5, 1H), 7.82 (s, 1H), 7.60 (s, 1H), 7.24 (d, *J* = 7.8, 1H), 6.30 (br, 1H), 4.00 (s, 3H), 3.33 (m, 1H), 1.26(m, 6H). ^13^C NMR(75 MHz, CDCl_3_) *δ*: 167.09, 158.82, 157.70, 155.33, 150.90, 131.50, 128.64, 120.99, 118.67, 116.89, 110.13, 105.98, 99.34, 52.53, 27.42, 22.96.

### ethyl 7-fluoro-1-hydroxy-8-isopropyldibenzo[b, d]furan-3-carboxylate (9b)

To a well stirred solution of compound 8 (0.95 g, 3.2 mmol) in MeOH (50 mL), was added diethylamine (DEA) (2 mL) in MeOH(20 mL) in dropwise at room temperature. After the addition, the mixture was stirred at ambient temperature for 12 hours. TLC and LC-MS examination showed that most of the starting material was converted into the target compound. The solvent was removed by rotary evaporation. The mixture was acidified to pH 1-2 with 5 mL 1N HCl. The residue was diluted with 50 mL of ethyl acetate. The mixture was washed with water and brine and dried over anhydrous Na_2_SO_4_. The precipitated product was filtered, and purified by recrystallization from a mixed MeOH/H_2_O solution (MeOH:H_2_O=3:1) to yield compound 9b (0.50g, yeild 69%). ESI-MS: [M - H]^−^ = 315,^1^H NMR(300 MHz, CDCl_3_) *δ*: 8.01 (d, *J* = 7.2, 1H), 7.83 (s, 1H), 7.59 (s, 1H), 7.23 (d, *J* = 7.2, 1H), 6.43 (br, 1H), 4.42(q, 2H), 3.33(m, 1H),1.43 (t, 3H), 1.27 (m, 6H), ^13^C NMR(75 MHz, CDCl_3_) *δ*: 165.95, 161.91, 157.51, 154.70, 153.44, 131.19, 129.61, 120.63, 119.24, 116.10, 110.19, 103.88, 100.16, 99.77, 61.43, 27.43, 23.18, 14.63.

### ethyl 1-(cyclopropylmethoxy)-7-fluoro-8-isopropyldibenzo[b, d]furan-3-carboxylate (10a)

compound 10a was prepared from compound 9b and (bromomethyl)cyclopropane as white crystal according to general method I.

ESI-MS: [M + H]^+^ = 371,^1^H NMR(300 MHz, CDCl_3_) δ: 8.06 (d, *J* = 7.8, 1H), 7.85 (s, 1H), 7.46 (s, 1H), 7.22 (d, *J* = 7.8, 2H), 4.34 (q, 2H), 4.14 (d, *J* = 6.9, 2H), 3.32 (m, 1H), 1.46 (t, 3H), 1.40 (m, 1H), 1.33 (d, *J* = 6.9, 6H), 0.71 (m, 2H), 0.52 (m, 2H), ^13^C NMR (75 MHz, CDCl_3_) δ: 166.54, 161.97, 158.71, 157.16, 157.13, 155.22, 155.03, 154.19, 131.46, 131.24, 129.34, 120.95, 120.86, 119.09, 119.07, 117.69, 106.15, 99.22, 98.83, 72.84, 61.28, 27.39, 22.89, 14.38, 10.25, 3.00.

### ethyl 7-fluoro-1-(hexyloxy)-8-isopropyldibenzo[b, d]furan-3-carboxylate (10b)

compound 10b was prepared from compound 9b and 1-bromohexane as white crystal according to general method I. ESI-MS: [M + H]^+^ = 401,^1^H NMR(300 MHz, CDCl_3_) δ: 8.01 (d, *J* = 7.5, 1H), 7.85 (s, 1H), 7.49 (s, 1H), 7.22 (d, *J* = 6.2, 1H), 4.42 (q, 2H), 4.26 (t, 2H), 3.32 (m, 1H), 1.97 (m, 2H), 1.63 (m, 2H), 1.42 (m, 4H), 1.26 (d, *J* = 6.9, 6H), 0.92 (t, 3H),^13^C NMR(75 MHz, CDCl_3_) δ: 166.56, 161.92, 158.66, 157.16, 157.33, 155.20, 155.01, 154.32, 131.43, 131.21, 129.37, 120.82, 120.73, 119.09, 119.07, 117.52, 106.05, 105.86, 99.21, 98.83, 68.51, 61.27, 31.64, 29.27, 27.31, 27.24, 22.91, 22.70, 14.38, 14.04.

### ethyl 1-(cyclohexylmethoxy)-7-fluoro-8-isopropyldibenzo[b, d]furan-3-carboxylate (10c)

compound 10c was prepared from compound 9b and (bromo methyl)cyclohexane as white crystal according to general method I.

ESI-MS: [M + H]^+^ = 413,^1^H NMR(300 MHz, CDCl_3_) δ: 8.04 (d, *J* = 7.5, 1H), 7.85 (s, 1H), 7.47 (s, 1H), 7.22 (d, *J* = 7.8, 2H), 4.40 (q, 2H), 4.08 (d, *J* = 5.4, 2H), 3.32 (m, 1H), 2.00 (m, 3H), 1.88 (m, 2H), 1.76 (m, 1H), 1.47 (t, 3H), 1.29 (d, *J* = 6.9, 6H),1.15 (m, 6H),^13^C NMR(75 MHz, CDCl_3_) δ: 166.58, 161.89, 158.63, 157.14, 157.11, 155.19, 155.01, 154.45, 131.41, 131.19, 129.38, 120.75, 120.66, 119.11, 119.08, 117.59, 106.01, 105.83, 99.20, 98.82, 73.79, 61.27, 38.06, 30.01, 27.10, 27.06, 26.54, 25.91, 22.91, 14.39.

### ethyl7-fluoro-8-isopropyl-1-((4-methoxybenzyl)oxy)dibenzo[b, d]furan-3-carboxylate (10d)

compound 10d was prepared from compound 9b and 1-(bromo methyl)-4-methoxybenzene as white crystal according to general method I.

ESI-MS: [M + NH4]^+^ = 454,^1^H NMR(300 MHz, CDCl_3_) δ: 7.99 (d, *J* = 7.5, 1H), 7.89 (s, 1H), 7.61 (s, 1H), 7.51 (d, *J* = 8.4, 2H), 7.22 (d, *J* = 7.5, 1H), 6.98 (d, *J* = 8.4, 2H), 5.31 (s, 2H), 4.40 (q, 2H), 3.87 (s, 3H), 3.28 (m, 1H), 1.43 (t, 3H), 1.29 (d, *J* = 6.9, 6H), ^13^C NMR(75 MHz, CDCl_3_) δ: 166.48, 159.58, 158.75, 157.20, 155.27, 153.88, 131.53, 131.31, 129.37, 128.94, 128.67, 120.94, 120.85, 118.96, 118.92, 117.84, 114.03, 106.49, 99.26, 98.88, 70.40, 61.32, 55.35, 27.37, 27.33, 22.83, 14.39.

### cyclopropylmethyl7-fluoro-1-(hexyloxy)-8-isopropyldibenzo[b, d]furan-3-carboxylate (11a)

compound 11a was prepared from compound 10b and (bromomethyl)cyclopropane as white crystal according to general method II and general method III.

ESI-MS: [M + H]^+^ = 427,^1^H NMR(300 MHz, CDCl_3_) δ: 8.01 (d, *J* = 7.5, 1H), 7.89 (s, 1H), 7.51 (s, 1H), 7.22 (d, *J* = 7.5, 1H), 4.42 (t, 2H), 4.20 (d, *J* = 7.2, 2H), 3.33 (m, 1H), 1.95 (m, 2H), 1.63 (m, 2H), 1.42 (m, 4H), 1.26 (d, *J* = 6.9, 6H), 0.92 (t, 3H), 0.62 (m, 2H), 0.42 (m, 2H), ^13^C NMR(75 MHz, CDCl_3_) δ: 166.70, 161.92, 158.66, 157.16, 157.13, 155.02, 154.35, 131.43, 131.21, 129.40, 120.83, 120.74, 119.08, 117.53, 106.12, 105.92, 99.23, 98.84, 70.23, 68.52, 31.65, 29.27, 27.31, 27.27, 25.99, 22.92, 22.71, 14.06, 9.96, 3.37.

### cyclopropylmethyl7-fluoro-8-isopropyl-1-((4-methoxybenzyl)oxy)dibenzo[b, d]furan-3-carboxylate (11b)

compound 11b was prepared from compound 10d and (bromomethyl)cyclopropane as white crystal according to general method II and general method III.

ESI-MS: [M + NH_4_]^+^ = 480,^1^H NMR(300 MHz, CDCl_3_) δ: 7.99 (d, *J* = 7.5, 1H), 7.92 (s, 1H), 7.62 (s, 1H), 7.51 (d, *J* = 8.4, 2H), 7.23 (d, *J* = 10.2, 1H), 6.97 (d, *J* = 8.7, 2H), 5.33 (s, 2H), 4.20 (d, *J* = 7.2, 3H), 3.87 (s, 3H), 3.28 (m, 1H), 1.29 (d, *J* = 6.9, 6H), 1.26 (m, 1H), 0.63 (m, 2H), 0.39 (m, 2H), ^13^C NMR(75 MHz, CDCl_3_) δ: 166.59, 159.57, 157.21, 157.18, 155.28, 155.09, 153.88, 131.52, 131.30, 129.40, 128.95, 128.67, 120.95, 120.85, 118.94, 117.84, 114.04, 106.56, 99.27, 98.88, 70.40, 70.06, 55.35, 27.38, 27.34, 22.83, 9.96, 3.38, 3.38.

### cyclohexylmethyl7-fluoro-1-(hexyloxy)-8-isopropyldibenzo[b, d]furan-3-carboxylate (11c)

compound 11c was prepared from compound 10b and (bromomethyl)cyclohexane as white crystal according to general method II and general method III.

ESI-MS: [M + H]^+^ = 469,^1^H NMR(300 MHz, CDCl_3_) δ: 8.01 (d, *J* = 7.5, 1H), 7.85 (s, 1H), 7.49 (s, 1H), 7.22 (d, *J* = 7.2, 2H), 4.28 (t, 2H), 4.17 (d, *J* = 6.3, 2H), 3.32 (m, 1H), 1.99 (m, 2H), 1.82 (m, 6H), 1.62 (m, 2H), 1.41 (m, 4H), 1.26 (m, 4H), 1.23 (d, *J* = 6.9, 6H), 1.13 (m, 6H), 0.88 (t, 3H), ^13^C NMR(75 MHz, CDCl_3_) δ: 166.61, 161.92, 158.66, 157.15, 157.12, 155.19, 155.01, 154.36, 131.44, 131.22, 129.42, 120.83, 120.75, 119.10, 119.07, 117.51, 105.97, 105.91, 99.20, 98.82, 70.36, 68.51, 37.32, 31.65, 29.81, 29.26, 27.32, 27.28, 26.39, 25.98, 25.73, 22.91, 22.70, 14.05.

### cyclohexylmethyl7-fluoro-8-isopropyl-1-((4-methoxybenzyl)oxy)dibenzo[b, d]furan-3-carboxylate (11d)

compound 11d was prepared from compound 10d and (bromomethyl)cyclohexane as white crystal according to general method II and general method III.

ESI-MS: [M + H]^+^ = 505, [M + NH_4_]^+^ = 522, ^1^H NMR(300 MHz, CDCl_3_) δ: 7.99 (d, *J* = 7.5, 1H), 7.88 (s, 1H), 7.60 (s, 1H), 7.51 (d, *J* = 8.4, 2H), 7.23 (d, *J* = 10.2, 1H), 6.91 (d, *J* = 8.7, 2H), 5.33 (s, 2H), 4.18 (d, *J* = 6.0, 3H), 3.86 (s, 3H), 3.29 (m, 1H), 1.72 (m, 6H), 1.29 (d, *J* = 6.9, 6H), 1.26 (m, 4H), 0.88 (m, 2H), ^13^C NMR (75 MHz, CDCl_3_) δ: 166.51, 162.02, 159.57, 157.20, 157.16, 155.26, 155.08, 153.88, 131.54, 131.32, 129.41, 128.91, 128.65, 120.95, 120.85, 118.96, 118.94, 117.83, 114.04, 113.78, 106.58, 106.42, 99.25, 98.87, 70.39, 55.34, 37.31, 29.81, 27.39, 27.36, 26.39, 25.74, 22.83.

### 1-(cyclopropylmethoxy)-7-fluoro-8-isopropyldibenzo[b, d]furan-3-carboxylic acid (11e)

compound 11e was prepared from compound 10a as white crystal according to general method II. ESI-MS: [M - H]^−^ = 341, ^1^H NMR(300 MHz, *d*_6_-DMSO) δ: 13.21 (br, 1H), 7.99 (d, *J* = 7.5, 1H), 7.77 (s, 1H), 7.63 (d, *J* = 7.8, 1H), 7.44 (s, 1H), 4.16 (d, *J* = 6.6, 2H), 3.22 (m, 1H), 1.21 (m, 2H), 1.29 (d, *J* = 6.9, 6H), 0.57 (m, 2H), 0.48 (m, 2H), ^13^C NMR(75 MHz, *d*_6_-DMSO) δ: 167.44, 161.73, 158.49, 156.97, 154.97, 154.77, 154.28, 131.48, 131.26, 131.03, 120.61, 120.52, 119.09, 117.50, 116.72, 106.94, 105.95, 100.28, 99.89, 72.72, 27.30, 23.12, 10.48, 3.19.

### PTP activity assay

Human recombinant PTP-MEG2, SHP2 and CDC25 were expressed in E. coli and purified by Ni-NTA affinity chromatography in our laboratory. The basic chemical reaction catalyzed by a phosphatase converts a phosphosubstrate into a dephosphorylated product and free phosphate which could be measured as a surrogate for phosphatase activity. pNPP(para-nitrophenyl phosphate) was used as phosphatase substrate which can be hydrolyzed by phosphatase to give para-nitrophenol. Subsequently, para-nitrophenol converts into para-nitrophenolate (pNP) with addition of sodium hydroxide stop solution. pNP is an intense yellow compound and could be measured at 405 nm using a spectrophotometer. To begin with, purified recombinant PTP-MEG2, SHP2 and CDC25 (0.05 μg) in 50 μL buffer with 50 mM citrate (pH 6.0), 0.1 M NaCl, 1 mM EDTA, and 1 mM dithiothreitol (DTT) and test compounds were added to each well of a 96-well plate. Blank was prepared by omitting enzyme and substituting an equivalent volume of buffer. After preincubation for 15 min at room temperature, 50 μL of reaction buffer with 2 mM pNPP was added and incubated at 37°C for 30 min. Then, the reaction was stopped by adding 10 μL 0.2 M sodium hydroxide and chilled on ice quickly. In addition, the amount of pNP was measured by detecting the absorption at 405 nm against blank. Finally, IC_50_ values were determined by analyzing the data using ORIGINPRO 8 software.

3D-common feature hypotheses generation and validation using the HipHop Method

**Figure 2:**
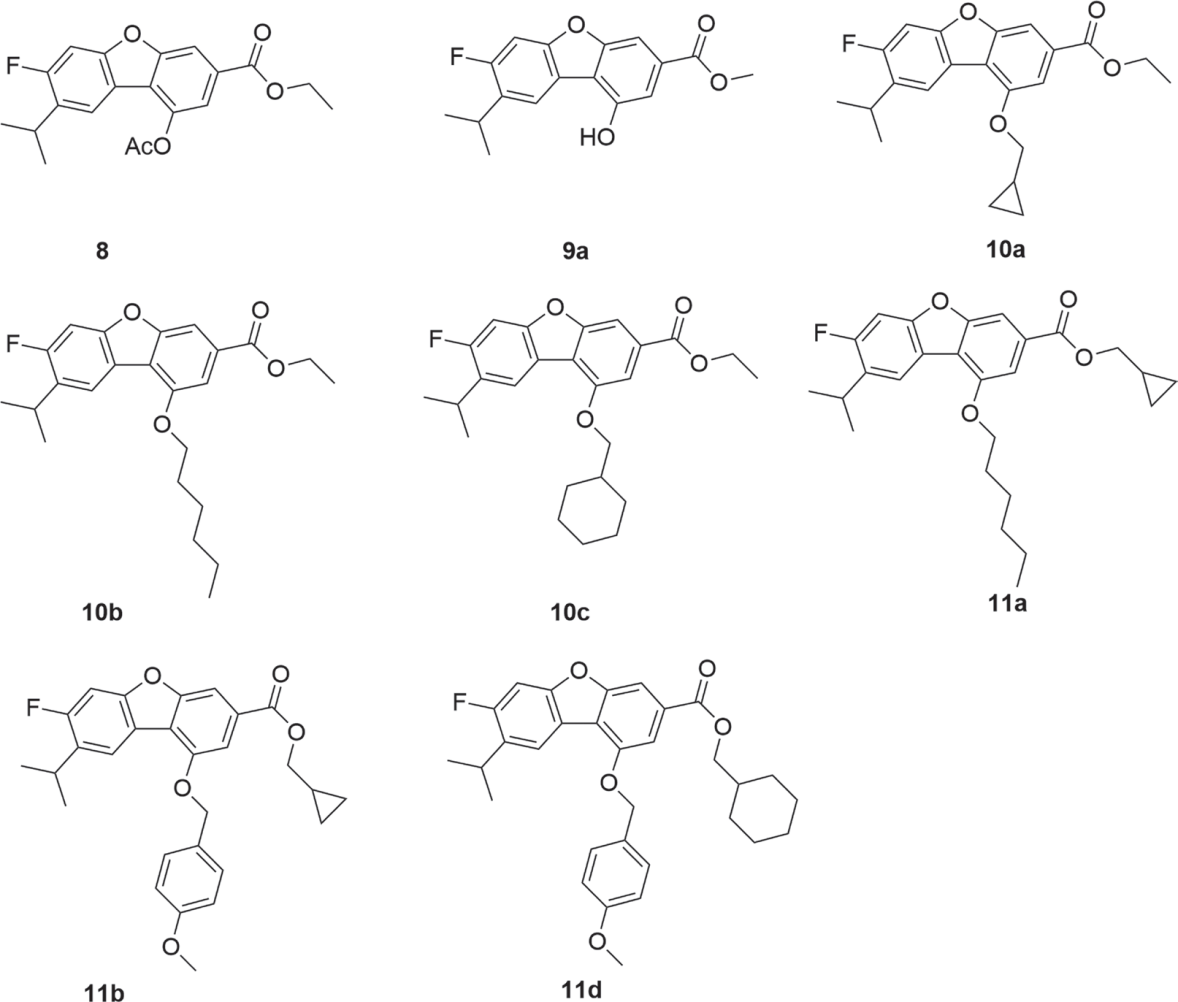
PTP-MEG2 inhibitors used in common feature pharmacophore generation

A data set of 8 compounds (Figure 2) for which *in vitro* inhibitory activities against the PTP-MEG2 enzyme synthesized in our lab were used as training set to develop a common feature 3D-pharmacophore model. Before the generation of pharmacophore hypotheses, the training set compounds were converted into 3D structure to generate diverse conformations using the Diverse Conformation Generation protocol implemented in Discovery studio v3.5. Per molecule will generate the maximum numbers of 200 conformations to ensure maximum coverage of the conformational space by using Best conformation model generation method with CHARMm force field [32] and Poling algorithm [33–36] module implemented in Discovery studio v3.5 was used to construct pharmacophore model in order to offer promising scaffolds for the development of novel and potent PTP-MEG2 inhibitors. The common feature pharmacophore generation used in this study was obtained by defining two properties-Pincipal and MaxOmitFeat of the ligands in the dataset that determined which molecules should be considered when building the pharmacophore space and which molecules should map to all or some of the features in the final pharmacophore. The Principal value of 2 and MaxOmitFeat value of 0 were assigned to the most active compounds (10a and 11d), which meant their structure and conformation would have the strongest influence in the model building phase. For the rest of the compounds, the Principal value of 1 and MaxOmitFeat value of 1 were assigned, which meant this molecule could partially map onto the hypothesis generated by the search procedure and all but one of the features in the generated pharmacophore must map to the compound. Selecting the chemical feature is one of the most important steps in generating pharmacophore. Due to the basic structures of the compounds and their proposed mechanism of action by Feature Mapping module from DS, four kinds of features including hydrogen-bond acceptor (HBA), hydrogen-bond donor (HBD), hydrophobic group (Hyd), and ring aromatic (RA) features were selected to initiate the pharmacophore hypotheses generation process. Moreover, the number of features of any particular type was allowed to vary from 0 to 5 for HBA, 0 to 5 for HBD, 1 to 5 for Hyd, and 1 to 5 for RA. All other parameters remained at their default settings.

Figure 2 PTP-MEG2 inhibitors used in common feature pharmacophore generation.

After automatic hypothesis generation, ten common features hypotheses with ranking scores were selected by the HipHop program. The ranking is a measure of how well the molecules map onto the proposed pharmacophores and the rarity of the pharmacophore model. However, the ranked first pharmacophore may not be the best pharmacophore model, and thus it is necessary to analyze all of them to determine which hypothesis was an accurate representation of the observed data. The derived pharmacophore map was validated based on Receiver operating characteristic (ROC) analysis to assess their abilities to selectively capture diverse PTP-MEG2 inhibitors from a large list of decoys. The decoys normally are selected from the zinc database, which are presumed to be similar to active ligands and be inactive against a target. The decoy set was generated using DecoyFinder [37]. A data set of 3 compounds for which *in vitro* inhibitory activities against the PTP-MEG2 enzyme synthesized in our lab were used as active molecules to search the decoy set by using the MACCS fingerprints and five physical descriptors [38–40]. The physical descriptors of a decoy are considered to be similar to those of an active ligand if the following conditions are met: (i) the molecular weight is within 25 Da of the active ligand; (ii) they contain the same number ± 1 of rotational bonds and HBDs, and the same number ± 2 of HBAs; and (iii) the Log *P value* is within 1.0 of the active ligand. The Tanimoto coefficients [27] between the MACCS fingerprints of each potential decoy and active molecule are then calculated. The Tanimoto coefficients between a potential decoy and each of the active molecules are not greater than 0.75. Thus, decoys are chemically different from any of the active molecules of the query. Finally, the decoys were generated such that each ligand has 30 decoys. The ROC testing set was screened by each pharmacophore for ROC analysis employing the “Best rigid search” option implemented in CATALYST, while the Maximum Omitted Features was set to -1. The default values for other parameters were kept constant. The ROC analysis validates pharmacophore model by analysis of sensitivity (Se) and specialty (Sp). In an optimal ROC curve, the value of the area under ROC curve (AUC) is 1; while random distributions cause the AUC value of 0.5. The AUC value needs to be between 0.5 and 1. The higher the value is, the better the discrimination is.

### Molecular docking

The Flexible Docking tool [41] embedded in Discovery Studio v3.5 was used as an efficient tool to monitor the interactions between ligands and target proteins. During the docking process, the selected side chains of amino acids and conformations of ligands are flexible. The preparation and refinement protocols for the protein receptor and all compound structures were performed on the Prepare Protein Wizard and Prepare Ligands modules embedded in the Discovery Studio v3.5. PTP-MEG2 (PDB ID: 4GE6) [20] was prepared by removing water, adding the hydrogen atoms, deleting alternate conformations, standardizing atom names and the ligands were prepared by the procedures of removing duplicates, enumerating isomers, tautomers, and ionization states [42] at a given pH range and generating 3D conformations. Define and Edit binding site tool embedded in Discovery Studio v3.5 was applied to calculate a binding site from a selected ligand. The P-loop (residues 514–521), the pTyr recognition loop (residues 331–338), and the Q-loop (residues 558–564) of PTP-MEG2 were selected to be used for creating protein conformations and side-chain refinement in the presence of the ligand [43]. All the investigated compounds were docked into the receptor pocket via the flexible protein docking model with the CDOCKER [44] scoring function to estimate the binding affinities.

### ADME prediction

ADME properties are a crucial aspect of clinical candidate quality. Approximately 39% of drugs were failing in development because of poor biopharmaceutical properties. With the high cost of development, this failure represented a major economic loss for the companies as well as the discovery of a new drug product was delayed. Lipinski's rule of five [45] is a rule of thumb to evaluate druglikeness or determine if a chemical compound would become a likely orally active drug in humans. The components of the rule are as follows: 1) No more than 5 hydrogen bond donors. 2) No more than 10 hydrogen bond acceptors. The increasing number of hydrogen bonds may reduce partitioning from the aqueous phase into the lipid bilayer membrane for permeation by passive diffusion. 3) A molecular mass less than 500 daltons. Increasing molecular weight (MW) reduces the compound concentration at the surface of the intestinal epithelium, which reduces absorption.4) An octanol-water partition coefficient log P not greater than 5. Increasing Log *P* also decreases aqueous solubility, thus reducing absorption. The polar surface area (PSA) is another determinant of fraction absorption. Structure properties determine physicochemical and biochemical properties, which ultimately determine pharmacokinetics and toxicity.

Calculations of important ADME properties of dibenzofuran derivatives were performed through Discovery Studio v3.5, USA (2013). With this software, a total of 15 categories of descriptors or molecular properties can be predicted, including the principal descriptors and pharmacokinetic properties. Molecular descriptors include 2D parameters (e.g., AlogP [46], molecular weight, number of aromatic ring, number of H-acceptors, number of H-donors, number of rings, number of aromatic rings, number of rotatable bonds, and molecular fraction polar surface area [47, 48]). The property analyses for van der Waals surface area of polar nitrogen and oxygen atoms (PSA), predicted aqueous solubility (Solubility) [49], human intestinal absorption[50, 51], blood brain barrier (ADMET_BBB_Level)[52], cytochrome p450 2D6 (ADMET_EXT_CYP2D6#Pre -diction) [53, 54], plasma protein binding (ADMET_EXT_PPB) [54–56]and hepatotoxicity (ADMET_EXT_Hepatotoxic#Prediction) [57, 58], were considered in the Discovery Studio v3.5 to evaluate the acceptability of the compounds.

## SUPPLEMENTARY FIGURES


