# Supplementary material for: Synthesis, bioactivity, 3D-QSAR studies of novel dibenzofuran derivatives as PTP-MEG2 inhibitors
Source: Oncotarget. 2017 Mar 27;8(24):38466–81. doi: 10.18632/oncotarget.16595 (PMC5503546; doi:10.18632/oncotarget.16595)
Supplement: Supplementary file 1 [file oncotarget-08-38466-s001.pdf]

Synthesis, bioactivity, 3D-QSAR studies of novel dibenzofuran derivatives as PTP-MEG2 inhibitors

Supplementary Materials

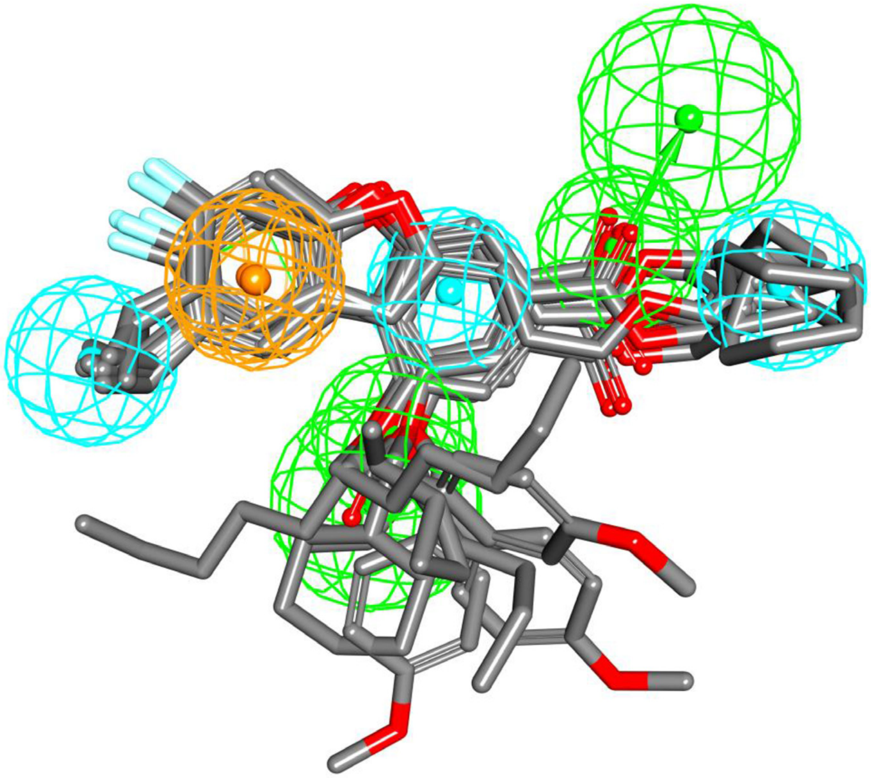

Supplementary Figure 1: Illustration to show the superimposed conformations of the eleven derivative compounds mapped to the best Hypogen model hypo-3-PTP-MEG2.

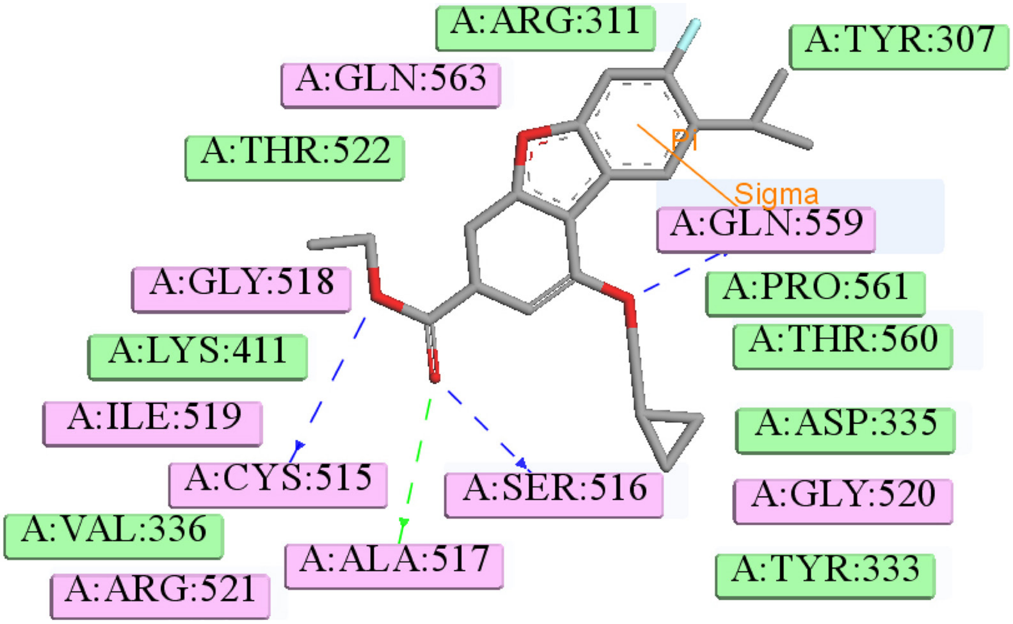

Supplementary Figure 2: The 2D diagram of PTP-MEG2-10a.
